# Analysis of Household Daily Water Consumption Dynamics in the Tropical Environment

**DOI:** 10.1155/2023/9956847

**Published:** 2023-08-03

**Authors:** Timothy O. Ogunbode, Victor O. Oyebamiji, Olumide A. Oluwole, John A. Akande

**Affiliations:** ^1^Environmental Management and Crop Production Unit, College of Agriculture, Engineering and Science, Bowen University, Iwo, Osun State, Nigeria; ^2^Department of Geography, Obafemi Awolowo University, Ile-Ife, Nigeria; ^3^Department of Geography, Nigerian Defence Academy, Kaduna, Nigeria

## Abstract

Understanding daily water use determinants is critical to sustainable water access and its efficient use at household level. To pursue this objective, primary data were generated through a survey of 276 respondents across the 5 quarters into which the city of Iwo is divided. 67.5% of the respondents were between 19 and 45 years old while 35.5% were between 46 and 65 years old and the remaining 3.9% comprised of those above 65 years. The results of factor analysis (FA) showed that 12 of the 40 factors analyzed were significant determinants of daily water use in homes. The 12 factors explained 85.794% of the observed variation in household daily water use. The factors in descending order were (i) closeness to water source; (ii) night time baths; (iii) household cooking; (iv) Sunday activities; (v) water demands of the dry season; (vi) morning time water use; (vii) household size; (viii) respondents' attitude; (ix) water availability in the dry season; (x) break in water flow; (xi) social events; and (xii) source of water. Further evaluation condensed the results into four categories, namely, (i) season-associated information; (ii) household-related information; (iii) time of the day; and (iv) water source-related information. The result of correlation analysis showed weak associations among 83.33% of the variables, indicating that each variable should be treated separately from others in the matter relating to daily variation in home water use. The study concluded that household-related information and time of the day (contributing 62.02% of the absolute explanation) are most significant to daily variation in water use at the household level and should be considered when prioritizing effective policies for water use and management.

## 1. Introduction

Sustainable, timely, and spatial access to quality water for domestic varied uses is an integral part of the global targets enlisted among the Sustainable Development Goals (SDGs) [1–3]. The reason for this is not far-fetched in view of the role of water in enhancing human livelihood globally. Water is required for both consumptive and non-consumptive purposes to keep living on. The uses of water transcend conventional uses such as drinking, bathing, and cooking. Ogunbode and Asifat [2] found that the prominent areas of water use in Southwestern Nigeria are drinking (100%), cooking (100%), bathing (100%), cloth washing (100%), dish washing (100%), toilet/sanitation (2.60%), and car wash (19.68%), thus indicating that water use is purely domestic, revealing poor sanitation and poor income according to these authors. In the contemporary times, the demand for water has become so expediently competitive that relevant stakeholders have become challenged by this trend especially in view of its finite nature. Further pressure on water demand was exacerbated with the COVID-19 pandemic and the associated diseases [1, 4]. The use of water for handwashing has been one of the ways of mitigating the spread of the disease globally. Climate change and its consequences on the natural environment have further worsened accessibility to water in areas that were water-stressed during pre-climate change era while those that were water-sufficient have to scout for the resource with a lot of cost implications on the part of man [5–7]. In order to ensure access to water at the household level, several submissions on factors touching on accessibility have been made and published [8–12]. However, little has been achieved on the dynamics of daily water use demand at household level, especially in the tropical regions of the world.

The focus on the tropical regions was because of the fact that the region is characterized with a lot of atmospheric dynamics that are often subjected to unpredictable variations [13–16]. The characterized variations in temperature, humidity, and rainfall have the potential to influence the pattern of daily utilization of water for domestic purposes [17–19]. The dynamics of daily atmospheric conditions in the tropical region have a significant impact on daily home water use. According to [20], tropical cyclones, such as hurricanes or typhoons, are common in tropical regions. These weather systems are associated with heavy rainfall, which can cause flooding, disruption of water supply infrastructure, and water quality distortion [21]. The aftermath of these cyclone activities may influence the pattern of water utilization in homes as potable water is being scouted for. Also, tropical region experiences high temperatures and humidity levels, which can aggravate evaporation rates [20]. This implies a higher demand for water in households for various uses such as drinking and bathing to maintain comfort. Apart from this, tropical regions often have distinct rainy and dry seasons. During the rainy season, there is an abundance of water from frequent precipitation [22]. However, in the dry season, water sources may become scarce. Water usage tends to be higher during the dry season, and so additional efforts are put to ensure that it is available to meet their daily needs. In addition, tropical regions often experience diurnal patterns in rainfall, with higher chances of rain and thunderstorms in the afternoon or evening [19]. Such variability can influence daily home water use because if rainfall occurs during the day, individuals may opt to collect rainwater for various purposes, reducing their reliance on other water sources [12, 23].

It is evident from the previous publications that water consumption at any time and space is dependent on factors like income, seasons, government policy, household gender composition, and household size [2, 22, 24, 25]. Detailed and comprehensive knowledge of daily water use variations at household level is significant to the predictability of weekly, monthly, and/or annual levels of water usage [3, 14, 26]. Such knowledge will enhance optimization of the use of water, prediction modelling, policy direction, and effective planning for sustainable living [5, 27, 28]. Tropical region is characterized with climate and weather variabilities which strongly influence water demand in time [20, 29, 30]. Weather descriptions in the tropics have often been described with terminologies such as cold, warm, hot, cloudy, rainy, dusty, and foggy, among others [3, 17]. These descriptions are salient to the level of water demand at any point in time [31, 32]. For instance, it is expected that less water will be utilized for drinking when the weather is cold, rainy, or cloudy while there is also likelihood that much volume of water will be used for bathing when the atmosphere is warm, dusty, or hot. Similar scenario applies when the day is rainy, and less is thought of washing cars while the reverse is the case when it is dry and dusty. To attain efficient use of water globally and, therefore, in the developing world importantly, a good and sound understanding of those factors that influence daily water demand at household level is required. This work is aimed at understanding the factors that influence variations in daily water use so as to enhance policy development and planning that will contribute to improved water access, its efficient use, and better water resource management. This aim was realized through (i) identification of the variables which impact on daily home water use; (ii) quantification of the variables identified; (iii) modelling household daily water use; and (iv) elucidating the possible association existing among the identified variables.

### 1.1. Theoretical Frameworks

The daily dynamics of household water use can be understood through various theoretical models and frameworks. Some of these frameworks are as follows.

Human behavior and decision-making framework require insights from behavioral theories. The theory of planned behavior (TPB), according to [33, 34], suggests that people's intentions and behaviors are influenced by their attitudes, subjective norms, and perceived behavioral control. TPB has been applied in water resource management, especially in water conservation at household level [33–37]. The application of this theory to household water use, according to [36], implies that individuals' decisions on when and how much water to use are partly and strongly shaped by their beliefs, social influences, and perceived capability to control their water consumption pattern. Time-use theory or time-allocation theory as propounded by Garry Becker in 1965 forms another framework to understand the dynamics of household water use. Time-use theory in this context emphasizes the allocation of time among different home activities, including household chores and water-related tasks [38, 39]. This theory explores how individuals prioritize and distribute their time throughout the day and how it impacts water use patterns [27, 37]. Factors such as the availability of water-related appliances, the time required for various water-related tasks, and the timing of activities can affect daily water use. Socioeconomic factors also play significant roles in modelling household water use dynamics. Economic theories, such as cost-benefit analysis [40, 41] and utility maximization [41, 42], provide insights into how individuals make decisions based on the perceived costs and benefits of water use. Income levels, water tariffs, access to infrastructure, and affordability of water-related technologies influence household water use patterns [12]. In addition to these, sociocultural factors also have strong influence on daily household water use pattern. Social norms, customs, and cultural practices determine individuals' attitudes and behaviors towards water use [43–45]. The World Health Organization [46, 47] also reiterated that culture can translate experience and assign value to knowledge, providing evaluation values for possible causes of human activity and sharing knowledge and prospects. For instance, in some cultures, water may be seen as a precious resource, leading to more conservation-oriented behaviors. Social networks and peer relationships can also affect the pattern of water use practices at either community or household levels. The availability and efficiency of water-related technologies and infrastructure influence daily water use dynamics [29]. The adoption of water-efficient fixtures, such as low-flow faucets and toilets, contributes to minimized consumption of water. The presence or absence of efficient piped water network, storage systems, and the availability of alternative water sources are significant influencers of domestic water use patterns. Environmental factors, such as climate conditions and water availability, interact with household water use dynamics [43]. The availability of water resources, seasonal variations in rainfall, and perennial and occasional water shortage or drought conditions influence the pattern of household daily water use [48]. Human consciousness of the environment and awareness of water conservation are significant determinants of individual behaviors.

These theoretical backgrounds provide a framework for understanding the complex interplay of individual choices, socioeconomic factors, cultural influences, technology, and environmental considerations that shape the daily dynamics of household water use.

## 2. Method of Study

### 2.1. Study Area

**Figure 1:**
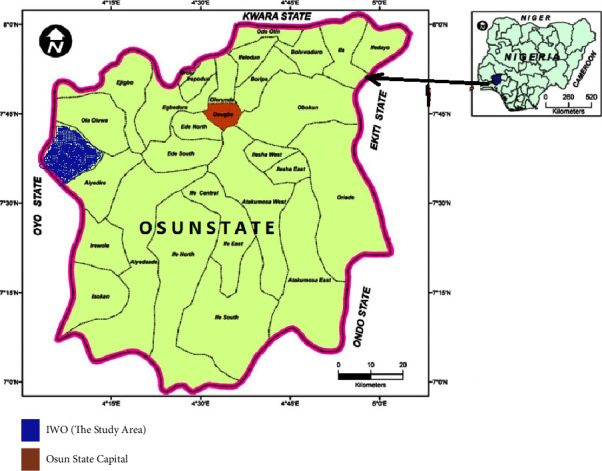
Map of Osun State showing the location of Iwo (inset: map of Nigeria showing the location of Osun State).

This research was conducted in Iwo town (Figure 1). Iwo is regarded as the headquarters of Iwo Local Government in Osun State in the southwestern part of Nigeria. It is located on 7°38′N and 4°11′E axis with a total area of 245 kilometer squared. Reniko et al. [23] had projected that the population of Iwo will be about 263,500 by 2022. The dominant climatic characteristic is that of tropic with two distinct seasons, namely, wet season running through March to October and dry season lasting from the month of November to February. Reniko et al. [23] discovered that annual rainfall in most tropical wet climate regions ranges between 1000 mm and 2000 mm with double maxima in July and September. Iwo habitants are predominantly farmers with high dependence on rainfall for agricultural practices. Apart from this, the dominant vegetation is tropical forest, comprising hardwoods such as obeche, walnuts, acacia, and eucalyptus, among others. In addition, the dominant agricultural practice is growing permanent and annual crops such as cocoa, kola nut, and oil palm and food crops such as yam and maize. However, the dry period in the year is often dominated with farming on the wetlands and along some riverbanks. Also, Iwo is drained by rivers such as Oba River, Ponyan River, Yanyanun River, and Aiba River. The town is seated on the Precambrian basement complex which is overburdened with soils such as clay and sandy soils.

### 2.2. Data Collection

Research data were generated through the administration of questionnaire in the study area. The sample frame comprised of married couples residing in each of the quarters of the town. Based on the available funds and the time available for the exercise, two hundred and eighty (280) copies of questionnaire were administered across randomly selected households from the five quarters into which the town is divided, namely, Molete, Gidigbo, Isale Oba, Oke-Oba, and Oke-Adan quarters. Iwo was chosen for this research in view of its unprecedented expansion and the associated increase in the need for social infrastructures, especially potable water at household level. Iwo has benefited from the gesture of the Federal Government of Nigeria with the citing of a Federal College of Education, apart from the increase in the size of the main market (Odo-Ori Market) and Bowen University (a faith-based private institution owned by Baptist Family in Nigeria). The survey considered female gender in each household because of their traditional roles in water provision in typical African homes [5]. According to [5, 49], females (mothers and their children in homes) are generally and culturally responsible for various home chores, including provision of water for various uses, in the African traditional homes. However, male gender was interviewed instead when the female head was not available or indisposed. The survey was carried out during the month of August which falls in the rainy season in the area. Structured questions focusing on various factors which exhibit influence on household water use as identified from the literature were highlighted for respondents' response. Such factors include seasonal variations, atmospheric conditions, time of the day, household parameters, attitudes, social events, and water source information.

### 2.3. Data Analysis

Both descriptive and inferential analyses of the data were carried out. Descriptive analysis includes the mean, percentages, and tabulation of the data while factor analysis (FA) was used to derive the principal variables influencing daily water use at household level by ranking. Correlation analysis (CA) was used to understand the association among the variables that were extracted by FA. The ranking by FA is significant to determine the degree of influence of each variable on household daily water use. The ranking factor, eigenvalue, was set at the minimum of 1.000. This implies that FA will present explanatory power and proportion of each variable involved in the analysis for those variables that weighted 1.000 at least. Those variables with less than the set minimum were not considered significant in the explanation of factors that influence household daily water use. Both FA and CA were carried out with the aid of an application, Statistical Package for Social Sciences (SPSS) Version 16.0.

## 3. Results and Discussion

The basic attributes and attitudes of the respondents are shown in Table 1.

About 67% of the entire respondents have post-primary qualification. Post-primary covers the education received after primary education but not up to tertiary level. Such levels include secondary education, technical education, and the like. The survey had preference for those respondents that will be able to complete the questionnaire on their own, hence the proportion of this category. The proportion of the respondents at primary level (17.0%) majorly includes mechanics (vehicle repairers), panel beaters, carpenters, micro-business operators, and drivers, among others. This group was either assisted by their neighbors or grown-up children or the research assistant himself/herself in completing the questionnaire. The inclusion of respondents who cannot read nor write in the survey was desirable since they too form part of the community where the research was conducted—they were assisted by the field assistants Also, the category of respondents with tertiary education was the least with 15.6% and it includes teachers, administrators, secretaries, and the like.

In terms of age, the survey focused on adult members of the study area, especially the married ones. The exercise, which was not gender-biased, showed that 60.5% were in the age range of 19 to 45 years, 35.5% in the range of 46 to 65 years, and 3.9% were above 65 years, as shown in Table 1. The survey deliberately avoided the last category in the household surveyed, but some retirees in that age range were accommodated when other preferred ones were not available.

The survey was largely dominated by the households of not more than five (5) in size with 64.5% of the entire respondents of 276, while those within the range of 6 to 10 members came second with 25.0%. Household size of 11 to 15 was 8.3% and those having more than 15 accounted for 2.2%. It was revealed that groundwater forms the major source of water to 89.1% of the respondents involved in the survey while a negligible proportion of 9.4% obtain water from pipe-borne water network and 1.5% claimed relying on surface water for their home use. The respondents have good access to water for their home uses as 96% of them claimed spending maximum of 30 minutes to get water (Table 1). This revealed the achievement of the global pursuit of improved access to water through Millennium Development Goals (MDGs) that ended in 2015, the sustainability of which is being currently pursued through Sustainable Development Goals (SDGs) [50].

### 3.1. Household Daily Water Demand Determinants

The results of Kaiser–Meyer–Olson (KMO) test of 0.875 and Bartlett's test of sphericity (BTS) (significant at *p* < 0.001) suggested that the dataset is factorable and adequate for factor analysis. The results of the analysis showing their respective rotated component matrix (RCM), eigenvalues, proportion of explanation of each of the 12 variables extracted percentage change in eigenvalues, and the percentage cumulative explanation are presented in Table 2. The twelve variables extracted were (i) closeness to water source; (ii) night time baths; (iii) household cooking; (iv) weekend activities; (v) water demands of dry season; (vi) morning time; (vii) household size; (viii) respondents' attitudes to water use; (ix) water availability in the dry season; (x) break in regular flow of water; (xi) social events; and (xii) source point of water. It is evident from the table that variable with the highest explanation is the closeness to water source (11.439%) while the variable with the least explanation is the source of water (whether surface, subsurface, or rainfall) (3.749%). The column which was generated in addition to the FA-generated values is the percentage change in the eigenvalue. This is significant to examine where the weights are more concentrated than the other on the ladder of the array of 12 extracted variables. The column showed that there was 24.93% change between weekend activities and water demanded in the dry season with the cumulative explanation of 47.652% in the daily variation in household water use in the study area out of the entire 85.794% explanation of the 12 extracted variables (i.e., 55.73% of the 100% explanation). This shows that closeness to water source (RCM = 85.5; eigenvalue = 4.576 and 11.439% contribution), night time baths (RCM = 90.7; eigenvalue = 3.902 and 9.756% contribution), household cooking (RCM = 84.7; eigenvalue = 3.864 and 9.660% contribution), weekend activities (RCM = 84.7; eigenvalue = 3.838 and 9.595% contribution), and water demands during the dry season (RCM = 88.3; eigenvalue = 2.881 and 7.202% contribution) had significant influence on the variation in the daily use of water at the household level.

Furthermore, the remaining 44.27% of the absolute explanation was given by the remaining seven (7) extracted factors. The change in the eigenvalue was still negligible (0.45%) between the fifth, water demands in the dry season (RCM = 88.3; eigenvalue = 2.881 and 7.202% contribution), and sixth variables, morning time (RCM = 84.2; eigenvalue = 2.868 and 7.171% contribution), and the sixth, household size (RCM = 84.7; eigenvalue = 2.760 and 6.901% contribution), and seventh variables, respondents' attitude (RCM = 90.1; eigenvalue = 2.171 and 5.427% contribution), but rose to 21.34% at the eighth variable. Thus, between the fifth and eighth variable, there was a contribution of 19.498% to the variations in the household daily water use. The last segment of the change in the weights of the variables was the final addition of 18.644% offered by the last five (5) variables, namely, respondents' attitude (RCM = 90.1; eigenvalue = 2.171 and 5.427% contribution), water availability during the dry season (RCM = 71.1; eigenvalue = 2.043 and 5.108% contribution), break in water flow (RCM = 82.9; eigenvalue = 2.035 and 5.087% contribution), social events (RCM = 87.0; eigenvalue = 1.880 and 4.700% contribution), and the source of water (RCM = 84.6; eigenvalue = 1.500 and 3.749% contribution).

It is clear that each of the extracted determinants varies in their respective contributions to the explanation of the daily variance in the household water use in the area studied. It is, therefore, imperative to show how each of the variables had manifested in the daily water use in homes.

#### 3.1.1. Closeness to Water Source *(Denoted as l)*

The respondents believed that their closeness to water source is significant to the pattern of their daily water demand as shown in Table 2. The authors of [51–53] had reported that one of the factors that influence household water use is the distance from the source of water. When respondents have short distance to cover in getting potable water for home use, there is possibility that more volume of water will be used in homes. The reverse is likely to be the case when the water source is far from the point of use. Yang and Wenyan [54] had recommended that the acceptable distance between hoes and water source should not take more than 30 minutes of trekking and not more than 500 meters; otherwise, such an environment will be categorized as water-stressed community. It was observed during the survey that only 96% of the respondents trek less than 30 minutes to get potable water for their homes as shown in Table 1.

#### 3.1.2. Time of the Day *(Denoted as t)*

The results, in Table 2, also indicated that daytime time characteristics significantly contributed to household daily water use in the study area. The daytime, such as morning, afternoon, and evening, exhibits impacts on daily water use in homes. The authors of [55, 56] had observed the impact of atmospheric dynamics on water supply which may also have effect on the availability of its use. It is evident in the result that water use in the morning is likely to be high since household members will need water for baths, cooking, and cleaning purposes as they set for the day's activities. However, the afternoon session of the day may not generally attract heavy use of water in homes because of the reduction in the household size and home chores. In the evening, the scenario has the tendency to shift upward as working-class members return back home and engage in some activities like night showers, cooking, and cloth washing and some other menial cleaning activities such as cleaning shoes and wear.

#### 3.1.3. Household Chores (Cooking) *(Denoted as c)*

Another strong determinant of household daily water use, as presented in Table 2, is the household chore including cooking. Other areas of water use in home such as cloth washing, sanitation, and baths may be put on hold for a while. The necessity of feeding members of the household may not be unavoidable on daily basis which then makes uses of water for cooking compulsory. In view of this, daily demand for water for cooking has that potential to influence the volume of water used daily in homes [57, 58].

#### 3.1.4. Weekend Activities *(Denoted as a)*

In addition, factor identified by FA is weekend activity (Table 2). Usually, weekend days such as Saturday and Sunday are often filled with various activities, some of which could have been suspended in the course of the week till the end of that week and some other activities that have been hitherto scheduled for weekend. Examples of such activities could include floor and drainage cleaning, general home cleaning and sanitation exercises, and massive cloth washing. In addition to these activities, ceremonies and festivals are often fixed for weekend days when those concerned will have enough time to attend to such as wedding and funeral programs, among others [59]. Religious activities, especially on Sundays, could also have increasing effect on the quantity of water required on weekend times.

#### 3.1.5. Water Demand during Dry Seasons (Denoted as d)

Table 2 also revealed that seasonal variations contributed to daily demand of water use at household level. The respondents were of the opinion that the variation in the season influences their demand for water on daily basis. It has been discovered that water supply in the dry season is albeit limited because yields from most groundwater sources become low and some rivers also become braided as a result of seizure in rainfall. It is, therefore, expected that water use during this period is often devoid of excessive use and undue wastage, most especially those environments that are often hit by the dry season [59]. At times, people in the study area need to travel distances before getting water for their home use, and so the habit of maximizing the little water obtained is unavoidable.

#### 3.1.6. Morning Time of the Day *(Denoted as m)*

Morning session of each day was also extracted as another strong determinant of daily water use in homes as shown in Table 2. This time of the day is usually characterized with various needs of water in homes. For instance, water is required for household members for bathing, cooking, washing, and other cleaning activities. Apart from these, the relevance of water for religious purposes like ablution cannot be overemphasized, especially in Muslim-dominated environments. The authors of [55, 56], in support of this discovery, noted that there was high volume of household water use in the morning and night time in Germany.

#### 3.1.7. Household Size *(Denoted as h)*

Household size was also discovered to have influence on household water use on daily basis as presented in Table 2. This discovery was not strange as several investigations have revealed the role household size plays when it comes to water demand. For instance, the authors of [2, 12, 23], among others, discovered that quantity of water use in homes varies with the size of the household. Thus, the larger the household is, the more likely the water is used in such homes for domestic activities.

#### 3.1.8. Respondents' Attitude *(Denoted as r)*

The power of attitudes to influence household water use has always been emphasized. For instance, the authors of [29, 33, 60] found that the variations found in the household water use were majorly attributed to the attitudes exhibited towards the resource in different households. Table 2 revealed that, out of the forty variables analyzed, attitude of water users was identified as a strong contributor to the variations experienced in domestic water use at household level. The perception of water users that water is a free good and so can be used anyhow is likely to stimulate excessive use of the resource in that household. The reverse is the case where the resource is diligently guided and treated as a resource that demands care and guidance in its use.

#### 3.1.9. Water Availability in the Dry Season *(Denoted as w)*

Furthermore, the results of the analysis in Table 2 showed that the availability of the resource in the dry season also contributed to the explanation for the variation noted in the use of water at household level. Two distinct seasons, wet and dry, were dominant in the study area. While wet season may last up to eight months with heavy downpour over the period, the dry season is characterized with no rainfall, dryness, and dusty condition. As a result of the characterized dryness and seizure of rainfall which is significant in the replenishment of other water sources, there is usually shortage of water, which may be serious in some other locations in the hinterland of Nigeria. The findings of also support this observation. Thus, the extraction of water availability in the dry season, as presented in Table 2, being one of the determining variables of household daily water use in Iwo, is not surprising.

#### 3.1.10. Breaks in Regular Water Flow *(Denoted as b)*

Breaks in regular water flow contributed to the explanation for the variation in home daily water use in Iwo, as shown in Table 2. It needs to be noted that Iwo township is endowed with different sources of water, namely, rainfall, surface, and subsurface sources. Pipe-borne water is also available in the town but very limited to a part of the town [22]. From this viewpoint, there is that tendency that few people that possibly have access to the source may possibly adapt themselves to the undependable flow of water from that source to ensure availability of water for their home use. The other scenario was the effect of seasonal fluctuations on groundwater outlets which could also exert break in the availability of the resource. The authors of also noted that irregular supply of water in some parts of Makurdi contributed to poor access to potable water.

#### 3.1.11. Social Events *(Denoted as e)*

The results of the analysis in Table 2 also identified social events as another determinant of the pattern of daily water demand in homes in the study area. Social engagements such as christening of newborn babies, wedding celebrations, house warming programs, and birthday celebrations, among others, are situations that could trigger rise in water use at household level. All these aforementioned events are usually occasional that often involve adequate preparations in advance because it entails unusual large number of people. The authors of [58, 59] lamented that social events and other cultural activities cannot be excluded in understanding domestic water uses, thus corroborating the observations in this research.

#### 3.1.12. Source of Water *(Denoted as s)*

The last of the factor extracted that explains the variations in the daily household water use as shown in Table 2 is the source of water to different homes. The authors of [2, 24] had revealed that the closer the source of water, the higher the volume of water consumed. Thus, a house that is well connected to pipe-borne water networks is likely to be prone to excessive use of water than those homes that only get water from the rivers in the forest or from very deep and unpowered hand-dug wells or where power supply is erratic. Ogunbode and Asifat [2] revealed that the source of water for household use should not exceed 30 minutes travelling from home for such a household to be described as water-rich.

On the basis of the above findings, the relationship between daily variations in household water use (vH) and the extracted variables can be expressed as

(1) $$\begin{array}{c} vH=f\left(l,t,c,a,d,m,h,r,w,b,e,s\right)\text{,} \end{array}$$

where *l* stands for closeness to water source; *t* for time of the day; *c* for house chores; *a* for weekend activities; *d* for demands of dry season; *m* for morning chores; *h* for household size; *r* for respondents' attitude; *w* for water availability in the dry season; *b* for break in the regular flow (supply) of water; *e* for social events; and *s* for source of water.

### 3.2. Further Assessment of the Explanatory Variables

Although twelve factors were found to be significant out of the forty variables involved in the analysis, a more critical evaluation of the extracted variables showed that four factors are more salient and major to daily variation in the household water use. The twelve factors are, therefore, condensed under the four principal factors (Table 3).

Table 3 shows household characteristics as having the greatest weight for explaining 26.687% of the variations in household daily water use, closely followed by time of the day that explained 26.522%. These two were followed by water-related information (20.463%), and far behind is the influence of seasonal changes (12.31%). The conclusion here is that much of the variation is dependent on household characteristics and time of the day, having 53.32% of the total explanation of 85.794% (61.88% of the absolute explanation). This observation also corroborated the findings in [25, 32].

The extracted variables, when condensed into four main components, the expression in (1) will result in equation:

(2) $$\begin{array}{c} vH=f\left(H,S,D,I\right)\text{,} \end{array}$$

where

 
*H* stands for household characteristics
 
*D* stands for day/atmospheric characteristics
 
*S* stands for seasonal changes
 
*I* stands for information on water.

### 3.3. Correlation between the Extracted Variables

In order to understand the possible association between the 12 extracted variables, correlation analysis was carried out. The result is shown in Table 4.

The results showed that the association between the twelve variables was weak, indicating that each of the extracted variables should be treated individually in the matters relating to their respective influence on daily variations in the household water use in the study area. However, two exceptional cases were noted, namely, associations between “*home chores in the dry season*” and “*closeness to water*” (*r* = 53.5%) and also between “*water use in the dry season*” and “*water demand in the dry season*” (*r* = 60.5%), both significant at *p* < 0.005. The two noted group associations revealed the significance of dry period on water use variation at household level.

### 3.4. Home Daily Water Use Variation Determinants at a Glance

**Figure 2:**
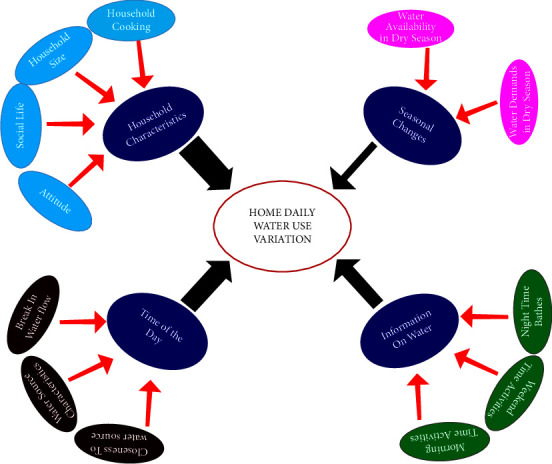
Schematic representation of the core determinants of household daily water use pattern (as designed by the authors).

Schematic representation of the determinants of household daily water use pattern is shown in Figure 2.

Figure 2 is a simplified schematic diagram of links between daily water use at household level, the principal components (condensed factors), and other extracted variables that explained the dynamics of daily use of water. At the core centre is the daily home water use variation being dependent on four principal components, namely, household characteristics, time of the day, information on water source, and seasonal changes. The magnitude of the influence of each of the principal components is shown in the width of the arrows linking each to the centre. The last rings surrounding each of the principal components are the attributes of each component as extracted by factor analysis. This graphical expression gives quick understanding of the relationships between the identified determinants and the household daily water use variation.

## 4. Conclusion and Recommendation

Investigation into what influences daily water use is desirable, if sustainable water access and its efficient use at household level will be realized as being pursued through the SDG 6. The results of FA showed that 12 factors out of the 40 analyzed were significant determinants of daily water use in homes, having given 85.794% of the explanation on the subject matter. Further analysis of the FA results showed four groupings of the extracted variables, namely, (i) season-associated information; (ii) household-related information; (iii) atmospheric condition/time of the day; and (iv) water source-related information. The results of correlation analysis showed weak associations among 83.33% of the variables, indicating individual consideration of each variable in the matter relating to daily variation in home water use. The study, therefore, concluded that household-related information and atmosphere/time of the day are strong determinants of daily variation in the pattern of water use at household level. It is, therefore, recommended that policies that hold household characteristics and atmospheric condition/time of the day on top priority on water supply be put in place and/or enhanced for sustainable water resource management.

## Figures and Tables

**Table 1:** Basic attributes of the respondents.

Categorization		Distribution	
Category no.	Attributes	Sample size	% of total in the category
A	Level of education		
	No formal education	0	0
	Primary	47	17.0
	Post-primary	186	67.4
	Tertiary	43	15.6

B	Age range		
	19 to 45	167	60.5
	46 to 65	98	35.5
	>65	11	3.9

C	Household size		
	<5	178	64.5
	6–10	69	25.0
	11–15	23	8.3
	>15	06	2.2

D	Sources of water		
	Pipe-borne	26	9.4
	Deep well/borehole	246	89.1
	Rivers/streams	4	1.5

E	Time taken to fetch water		
	<10 minutes	93	33.7
	11–20 minutes	127	46.0
	21−30 minutes	45	16.3
	>30	11	4,0

**Table 2:** Report of factor analysis on the extracted variables.

Determinant variables	RCM	Eigenvalue	% change in eigenvalue	% explained	% cumulative
Closeness to water source	85.5	4.576	0	11.439	11.439
Night time baths	90.7	3.902	14.73	9.756	21.194
Household cooking	84.7	3.864	0.97	9.66	30.855
Weekend activities	84.7	3.838	0.67	9.595	40.45
Demands of the dry season	88.3	2.881	24.93	7.202	47.652
Morning time	84.2	2.868	0.45	7.171	54.823
Household size	84.7	2.76	3.77	6.901	61.723
Respondents' attitude	90.1	2.171	21.34	5.427	67.15
Water availability in the dry season	71.1	2.043	5.9	5.108	72.259
Break in water flow	82.9	2.035	0.39	5.087	77.346
Social events	87	1.88	7.62	4.7	82.045
Source of water	84.6	1.5	20.21	3.749	85.794

**Table 3:** Condensed variables affecting daily home water use.

S/no.	Principal components	Subcomponents	% weighted	Total weighted (%)
1	Seasonal changes	Water demands of the dry season	7.202	12.31
		Water availability in the dry season	5.108	

2	Household characteristics	Household cooking	9.660	26.687
		Household size	6.901	
		Social life	4.700	
		Attitude	5.427	

3	Atmosphere/time of the day	Night time baths	9.756	26.522
		Weekend days	9.595	
		Morning time	7.171	

4	Information on water	Closeness to water source	11.439	20.275
		Water source characteristics	3.749	
		Break in water flow/supply	5.087	

			Grand total	85.794

**Table 4:** Results of correlation analysis between the extracted variables.

Variable names	Closeness to water	Night bath	Demand of dry season	Sunday activities	Home chores dd	Morning needs	Household size	Dry season use	Respondents' view	Break in flow	Social event	Source
Closeness to water	1											
Night bath	0.198	1										
Demands of ds	0.096	0.157	1									
Sunday activities	0.111	028	061	1								
Home chores dd	0.535	0.166	0.179	0.207	1							
Morning needs	0.070	0.166	0.179	016	0.131	1						
Household size	−069	0.053	0.130	0.090	0.245	130	1					
Dry season use	−182	−309	−609	0.305	0.158	−306	−192	1				
Respondents' view	−057	−290	−146	0.069	−107	−107	0.217	−204	1			
Break in flow	−200	−307	−300	374	−173	043	0.144	0.209	0.329	1		
Social event	−046	−245	−118	−137	−087	−087	−086	−203	−071	−247	1	
Source	0.001	0.011	0.003	0.065	0.002	−0.050	−050	−095	−010	−005	−040	0.91

Significant at *p* < 0.005.

## Data Availability

The data used to support the findings of this study are included within the article.
